# Ameliorative Effects of Taurine Against Methimazole-Induced Cytotoxicity in Isolated Rat Hepatocytes

**DOI:** 10.3797/scipharm.1205-16

**Published:** 2012-08-06

**Authors:** Reza Heidari, Hossein Babaei, Mohammad Ali Eghbal

**Affiliations:** 1 Drug Applied Research Center, Tabriz university of medical sciences, 51656-65811 Tabriz, Iran.; 2 Department of pharmacology and Toxicology, Faculty of Pharmacy, Tabriz University of Medical Sciences, 51656-65811 Tabriz, Iran.

**Keywords:** Methimazole, *N*-Methylthiourea, Taurine, Hepatotoxicity

## Abstract

Methimazole is used as an antithyroid drug to control the symptoms of hyperthyroidism and maintain patients in a euthyroid state. Administration of this drug is associated with agranulocytosis and hepatotoxicity, which are the two most significant adverse effects. The present investigation was conducted to study the protective role of taurine against cytotoxicity induced by methimazole and its proposed reactive intermediary metabolite, *N*-methylthiourea, in an *in vitro* model of isolated rat hepatocytes.

At different points in time, markers such as cell viability, reactive oxygen species (ROS) formation, lipid peroxidation, mitochondrial membrane potential, and hepatocyte glutathione content were evaluated.

Treating hepatocytes with methimazole resulted in cytotoxicity characterized by the reduction in cell viability, an increase in ROS formation and lipid peroxidation, mitochondrial membrane potential collapse, and a reduction in cellular glutathione content. Furthermore, a significant amount of oxidized glutathione (GSSG) was formed when rat hepatocytes were treated with methimazole. *N*-methylthiourea toxicity was accompanied by a reduction in cellular GSH content, but no significant changes in lipid peroxidation, ROS formation, GSSG production, or changes in mitochondrial membrane potential were detected. Administration of taurine (200 μM) effectively reduced the toxic effects of methimazole or its metabolite in isolated rat hepatocytes.

## Introduction

Methimazole is one of the most convenient drugs used in the treatment of hyperthyroidism and the reduction of thyroid function before surgery [1]. On the other hand, many authors have reported hepatotoxicity as a deleterious effect accompanying the use of methimazole [2, 3]. *N*-methylthiourea is a proposed metabolite for methimazole which is suspected to be responsible for methimazole-induced hepatotoxicity [4].To prevent methimazole-induced toxicity, no particular protective agents have been reported.

Taurine is a conditionally essential amino acid containing a sulfonic acid group with several physiological roles [5]. There are many reports on taurine’s protective effects against different chemically-induced hepatotoxicity [6–11]. Furthermore, taurine has shown protective effects in clinical situations such as diabetes [12] and pancreatitis [13]. It has been reported that this amino acid could act as an antioxidant in biological systems [14]. Hence, the protective effects of taurine could be due to the antioxidant capability of this amino acid. Being an antioxidant, it also has the ability to scavenge the reactive oxygen species, attenuate lipid peroxidation, and consequently stabilize biological membranes [15]. The goal of the present study was, therefore, to investigate the beneficial role of taurine against cytotoxicity induced by methimazole and its reactive metabolite. Cellular damage was evaluated by measuring the percent of viable cells using the trypan blue exclusion test. The possibility of reactive oxygen species (ROS) formation and lipid peroxidation was assessed and the effect of methimazole and its metabolite on cellular defense mechanisms such as intracellular glutathione was studied. Furthermore, the effect of methimazole on hepatocyte mitochondria was evaluated.

## Results and Discussion

**Fig. 1.**
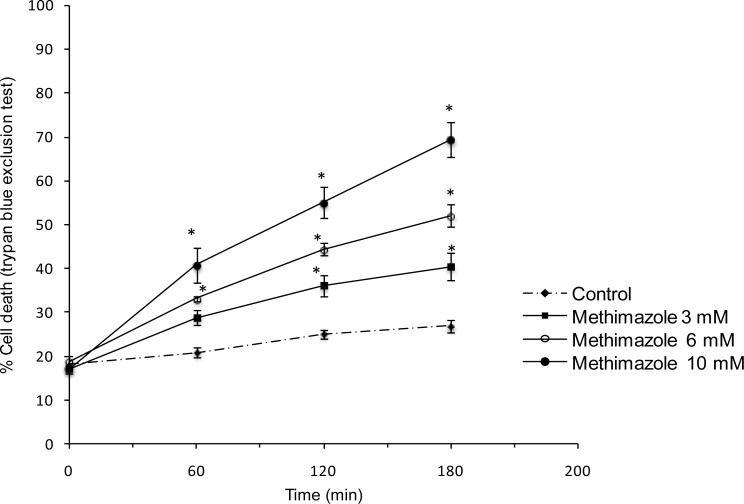
Dose-response of methimazole-induced cytotoxicity in rat hepatocytes. Data represent Mean±SE for at least three independent experiments. * P<0.05 indicates significant difference as compared to control group.

**Fig. 2.**
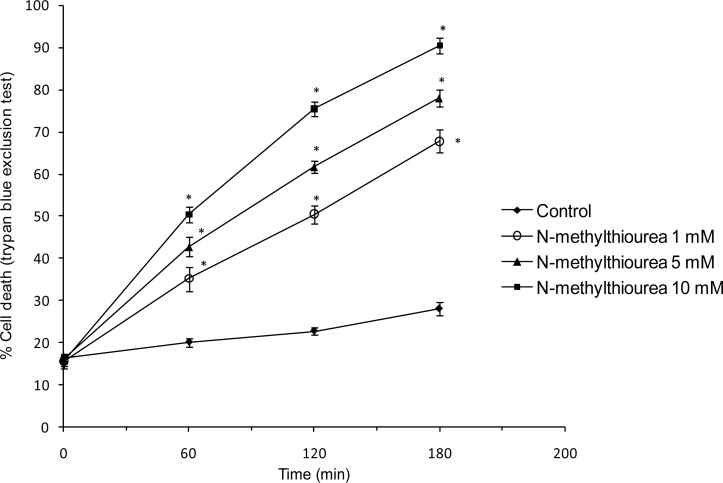
*N*-methylthiourea cytotoxicity in isolated rat hepatocytes. Data given as Mean±SE for at least three separate experiments. * P<0.05 shows significant difference as compared to control group.

Methimazole toxicity in rat hepatocytes was concentration-dependent with 10 mM methimazole causing about 50% death in 2 h (LC_50_) as measured by the trypan blue exclusion assay (Figure 1). *N*-methylthiourea caused cell death in lower concentrations than the parent drug. The LC_50_ dose for *N*-methylthiourea was found 1 mM (Figure 2).

**Fig. 3.**
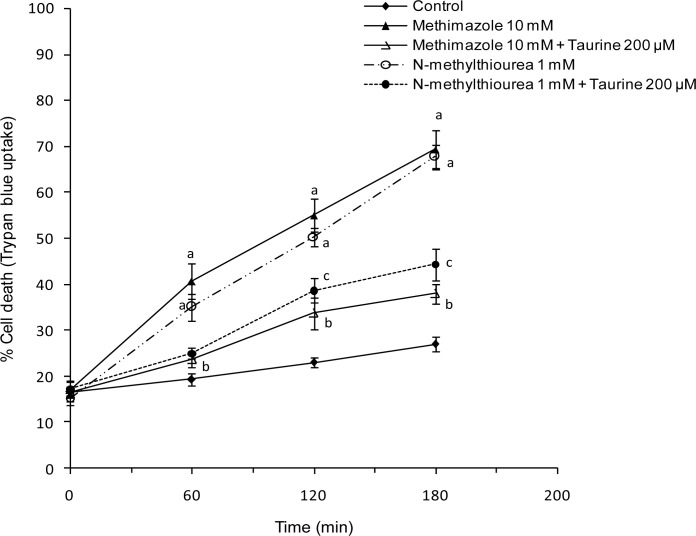
Protective effect of taurine against cell death induced by methimazole *N*-methylthiourea in isolated rat hepatocytes. Taurine (200 μM) was added 30 minutes before other agents. Data represent Mean±SE for three separate experiments. ^a^: Significantly different from control group (P<0.001). ^b^: Significantly different from methimazole-treated group (P<0.01). ^c^: Significantly different from *N*-methylthiourea treated group (P<0.01).

An optimum effective dose of taurine that provided appropriate protection was found (200 μM). Hepatocytes were treated with taurine 30 minutes before adding methimazole or *N*-methylthiourea. It was found that taurine effectively prevented cell death induced by methimazole or *N*-methylthiourea (Figure 3).

Markers such as ROS formation, lipid peroxidation, cellular glutathione content, and mitochondrial membrane potential were assessed to investigate the mechanism by which taurine protected hepatocytes against methimazole-induced toxicity and to elucidate the cause of cell death induced by methimazole.

**Fig. 4.**
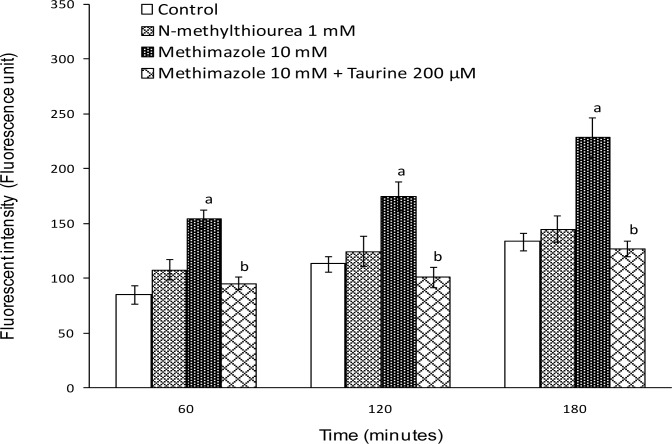
Methimazole-induced ROS formation in isolated rat hepatocytes and the protective effect of taurine. Taurine (200 μM) was added 30 minutes before other agents. Data are given as Mean±SE for at least three separate experiments. ^a^: Represents significant difference as compared to control group (P<0.05). ^b^: Represents significant difference as compared to Methimazole-treated group (P<0.05).

A significant amount of reactive oxygen species were formed when hepatocytes were treated with methimazole, but *N*-methylthiourea did not cause any ROS formation (Figure 4). Pretreatment of isolated hepatocytes with taurine reduced methimazole-induced ROS formation (Figure 4).

As previously mentioned, it has been found that taurine could act as an antioxidant and a radical scavenger [14]. Hence, the reactive oxygen species formed during methimazole metabolism were scavenged by taurine and this may have had a role in its protective effects in methimazole cytotoxicity. *N*-methylthiourea was unable to increase ROS formation in rat hepatocytes (Figure 4). This could indicate that other methimazole metabolites other than *N*-methylthiourea are responsible for ROS formation induced by this drug. In previous investigations, it has been shown that the *N*-methylthiourea produced during methimazole metabolism is further metabolized to reactive metabolites, which are capable of causing cytotoxicity [16, 17]. The protective effect of taurine against *N*-methylthiourea cytotoxicity in rat hepatocytes could be capable of inactivating these reactive metabolites.

**Fig. 5.**
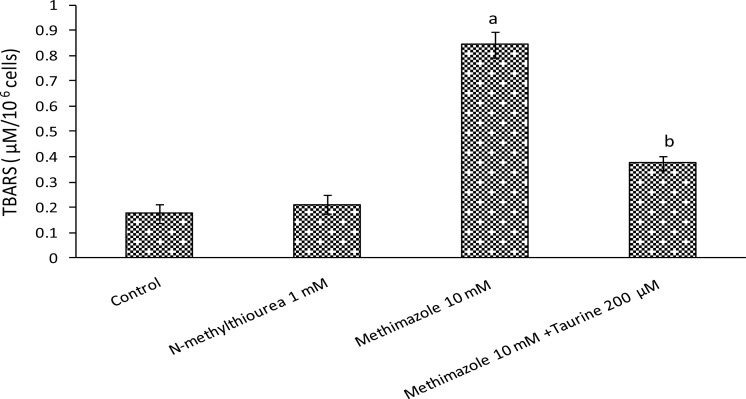
Methimazole-induced lipid peroxidation and the effect of taurine. Data given as Mean±SE for three separate experiments as measured after 120 minutes of incubation time. Taurine (200 μM) was added 30 minutes before other agents. ^a^: Indicates significant difference as compared to control group (P<0.001). ^b^: Indicates significant difference as compared to methimazole-treated group (P<0.001).

Lipid peroxidation is usually one of the consequences of ROS formation and oxidative stress in biological systems [18]. Methimazole caused lipid peroxidation in isolated rat hepatocytes, but there was no difference between *N*-methylthiourea-treated groups and control groups in lipid peroxidation (Figure 5) which is not unusual, as *N*-methylthiourea did not cause any significant ROS formation. It was found that taurine effectively prevented lipid peroxidation in methimazole-treated cells (Figure 5). The role of taurine in attenuating the lipid peroxidation induced by methimazole may have been due to its effects in modulating the oxidative stress caused by this drug. As shown in Figure 5, *N*-methylthiourea did not cause lipid peroxidation in rat hepatocytes and this might suggest that other methimazole metabolite(s) were responsible for the lipid peroxidation induced by this drug.

**Fig. 6.**
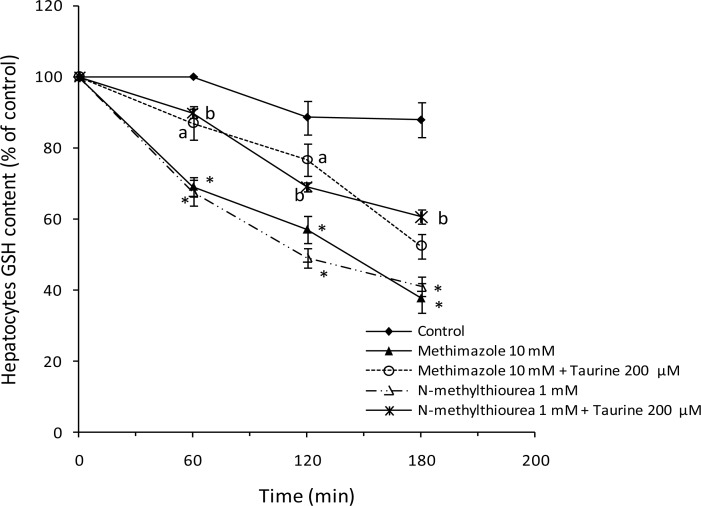
Effect of methimazole and *N*-methylthiourea on hepatocytes GSH content and the role of taurine. Data are shown as Mean±SE for three independent experiments. Hepatocytes were preincubated with taurine (200 μM), 30 minutes before adding other agents. ^*^: Represents difference between control and drug treated groups (P<0.001). ^a^: Significant as compared to methimazole-treated group (P<0.05). ^b^: Significant as compared to *N*-methylthiourea treated group (P<0.05).

The effect of methimazole and *N*-methylthiourea on hepatocyte glutathione reservoirs was studied. Both methimazole and *N*-methylthiourea caused a significant reduction in cellular glutathione content (Figure 6). Glutathione is a vital molecule that prevents the deleterious effects of oxidative stress. Furthermore, glutathione conjugates with different xenobiotics and detoxifies them [19]. A reduction in cellular glutathione by methimazole or its metabolite leaves hepatocytes defenseless against different stresses such as ROS formation. This may have a role in methimazole-induced toxicity in hepatocytes. Taurine can react with free radicals or reactive metabolites produced during methimazole metabolism, and hence prevent glutathione consumption and a reduction in hepatocytes. Preventing the depletion of glutathione reservoirs could be another mechanism by which taurine attenuates the toxicity of methimazole or its reactive metabolites in hepatocytes.

**Fig 7.**
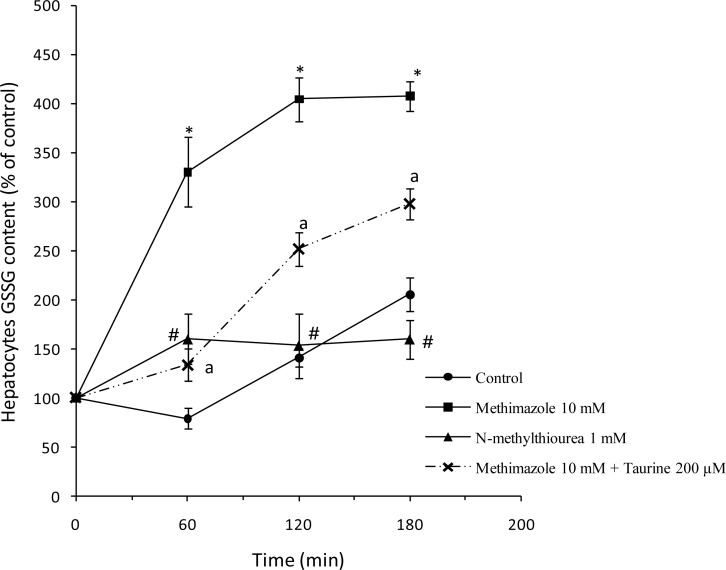
Cellular oxidized glutathione (GSSG) content after treating isolated rat hepatocytes with methimazole or *N*-methylthiourea. ^*^: Significant difference as compared to control group (P<0.001). ^a^: Significant difference as compared to the methimazole-treated group (P<0.01). ^#^: *N*-methylthiourea caused no significant changes in cellular GSSG content as compared to the control group.

GSH reduction caused by methimazole was accompanied with a significant increase in the amount of oxidized glutathione (GSSG) in rat hepatocytes (Figure 7) indicating that GSH depletion might be mainly due to its oxidation to GSSG. Treating rat hepatocytes with taurine significantly reduced the amount of GSSG formed after methimazole administration (Figure 7). It was found that there was no significant difference between the *N*-methylthiourea-treated group and control group in GSSG levels (Figure 7). The increase in cellular GSSG suggests the occurrence of oxidative stress. Hence, methimazole-induced elevation in cellular GSSG contents may be due to its ability in inducing ROS formation and oxidative stress in hepatocytes. Since there was no oxidative stress in *N*-methylthiourea-treated cells (Figure 4), it is justifiable that the level of GSSG in these cells was not significantly different from that in the control groups (Figure 7). Reducing the level of oxidized glutathione formed during methimazole toxicity could be attributed to the role of taurine in preventing oxidative stress and the oxidation of GSH to GSSG.

**Fig. 8.**
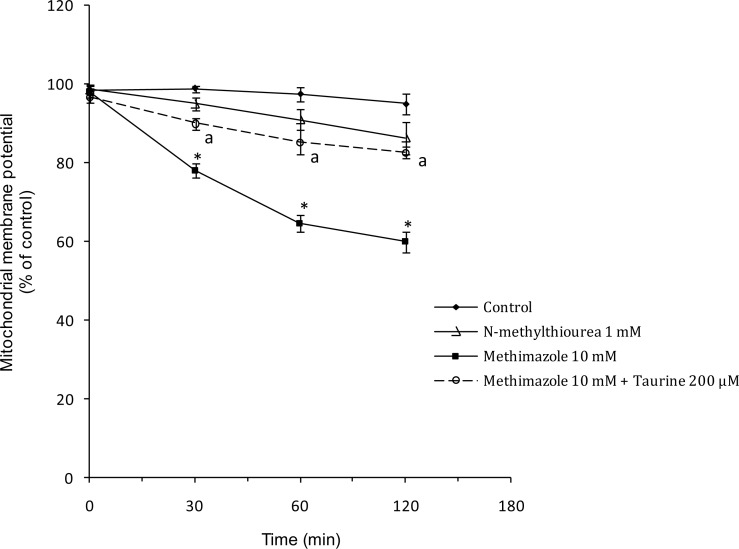
Effect of methimazole and *N*-methylthiourea on mitochondrial membrane potential and the protective role of taurine. ^*^: Different as compared to control (P<0.001). ^a^: Different as compared to methimazole-treated group (P<0.05).

The effect of methimazole and its metabolite on the mitochondria as the energy-producing and key organelle of hepatocytes was evaluated. It was found that methimazole caused mitochondrial membrane potential reduction (Figure 8). *N*-methylthiourea caused no significant changes in mitochondrial membrane potential (Figure 8). Taurine attenuated the reduction in mitochondrial membrane potential caused by methimazole (Figure 8). This effect may be due to the ability of taurine in scavenging the reactive metabolites produced during methimazole metabolism.

Our data show that methimazole cytotoxicity in rat hepatocytes is accompanied by an increase in reactive oxygen species, which suggests that methimazole induces hepatotoxicity through oxidative stress. The induction of oxidative stress by xenobiotics is usually accompanied by lipid peroxidation and a reduction in cellular glutathione reservoirs as it was found in methimazole toxicity. The collapse in mitochondrial membrane potential seems to be another mechanism through which methimazole causes toxicity. Mitochondrial damage could be a consequence of oxidative stress in cells [20]. The methimazole metabolite did not induce oxidative stress in hepatocytes, but the reduction in glutathione reservoirs showed that this metabolite is reactive or may produce reactive species.

It has been shown that *N*-methylthiourea is metabolized to sulfenic acids, which are very reactive [16, 17]. These reactive metabolites could conjugate with glutathione and get detoxified or may react with other targets such as proteins and induce cellular damage. The protective effects of taurine in *N*-methylthiourea-induced toxicity might be due to conjugating these reactive metabolites.The effect of taurine against the cytotoxicity induced by methimazole and its metabolite makes this amino acid the subject of further studies for developing an effective protective agent against different kinds of drug-induced liver damage.

## Experimental

### Chemicals

Taurine, (4-(2-hydroxyethyl)-1-piperazine-ethanessulfonic acid (HEPES), 2-vinyl pyridine, triethanolamine, and oxidized glutathione (GSSG) were obtained from Acros (New Jersey, USA). Methimazole was purchased from Medisca pharmaceutique incorporation (Montreal, Canada). Albumine bovine type was purchased from the Roche diagnostic corporation (Indianapolis, USA). Rhodamine 123, 5,5′-dithiobis(2-nitrobenzoic acid) (DTNB), 2′,7′-dichlorofluorescin diacetate, glutathione reductase from baker’s yeast, β-Nicotinamide adenine dinucleotide (NADPH), and collagenase from clostridium histolyticum, were obtained from Sigma Aldrich (St. Louis, USA). Ethylene glycolbis(2-aminoethylether)-*N*,*N*,*N*′,*N*′-tetraacetic acid (EGTA), *N*-methylthiourea, trichloroacetic acid (TCA) and trypan blue were obtained from Merck (Darmstadt, Germany). Thiobarbituric acid (TBA) was obtained from SERVA (Heidenberg, New York). All salts used for preparing buffer solutions were of analytical grade and obtained from Merck (Darmstadt, Germany).

### Experimental animals

Male Sprague-Dawley rats (250–300 g) were kept in ventilated plastic cages over hardwood bedding. There was an ambient temperature of 21–23 °C with a 50–60% relative humidity. Animals were fed a normal chow diet and water *ad libitum*. Collagenase perfusion via the portal vein was used to isolate rat hepatocytes as described previously [21]. This technique is based on liver perfusion with collagenase after the removal of calcium ions (Ca^2+^) with a chelator (EGTA 0.5 mM). The livers were perfused with different buffer solutions through the portal vein. The collagenase-containing buffer solution destructed liver interstitial tissue and caused hepatocytes to be easily isolated in the next steps. Isolated hepatocytes (10 mL, 10 6 cells/mL) were incubated in the Krebs-Henseleit buffer (pH 7.4) in continuously rotating 50 mL round bottom flasks, under an atmosphere of carbogen gas (95% O_2_ and 5% CO_2_) in a 37 °C water bath.

### Cell viability

After the hepatocyte isolation process, cell viability was assessed by the extent of plasma membrane intactness as determined by the trypan blue (0.1%, w/v) exclusion test microscopically [22]. Only the cell preparations with a viability above 85% were used. Hepatocyte viability was determined at scheduled time intervals during the experiment. In all experiments, taurine was added 30 minutes before other agents.

### Mitochondrial membrane potential assay

The fluorescent dye, rhodamine 123 accumulated in intact mitochondria by facilitated diffusion. When the mitochondrion was damaged and the mitochondrial membrane potential was reduced, there was no facilitated diffusion and the amount of rhodamine 123 in the supernatant increased. At the given times, 1 mL samples of the cell suspension were taken and centrifuged at 1000 rpm for 1 minute. Then the cell pellet was resuspended in 2ml of Krebs-Henseleit buffer containing 1.5 μM rhodamine 123 and incubated at 37 °C in a water bath with gentle shaking. Hepatocytes were separated by centrifugation at 3000 rpm for 1 minute and the amount of rhodamine 123 appearing in the incubation medium was measured fluorimeterically using a Jasco FP-750 fluorescence spectrophotometer (490 nm excitation and 520 nm emission wavelengths). The capacity of mitochondria to take up the rhodamine 123 was calculated as the difference in fluorescence intensity between the control and treated cells and was expressed as the percentage of the control [23].

### Lipid peroxidation

During the degradation of lipids, thiobarbituric acid reactive substances (TBARS) such as malondialdehyde (MDA) are formed [24]. Lipid peroxidation in hepatocytes was determined by measuring the amount of TBARS. Briefly, 1 ml aliquots of hepatocyte cell suspensions (10^6^ cells/ml) were treated with 250 μL trichloroacetic acid (70% w/v) and centrifuged at 3000 rpm for 15 min. Then 1mL of thiobarbituric acid (0.8% w/v) was added and boiled for 20 minutes. The developed color was read at 532 nm using a Pharmacia Biotech Ultrospec 2000 spectrophotometer. TBARS formation was expressed as μM TBARS 10^6^ cells ^−1^.

### Cellular GSH/GSSG content

The hepatocyte glutathione (GSH) content was determined by the method of Ellman [25]. A 1 ml aliquot of the cell suspension (10^6^ cells) was taken and 2 ml of 5% TCA was added and centrifuged. Then 0.5 ml of Ellman’s reagent (0.0198% DTNB in 1% sodium citrate) and 3 ml of the phosphate buffer (pH 8.0) were added. The absorbance of the developed color was measured at 412 nm using a Biotech Pharmacia Ultrospec^®^ 2000 spectro-photometer. Cell samples were reduced with potassium borohydride (KBH_4_) to prevent GSH oxidation during the experiment [26]. The enzymatic recycling method was used to assess the hepatocyte oxidized glutathione (GSSG) level [27], where cellular GSH content was covalently bonded to 2-vinylpyridine at first. Then the excess 2-vinylpyridine was neutralized with thriethanolamine, and GSSG was reduced to GSH using the glutathione reductase enzyme and NADPH. The amount of GSH formed was measured as already described for GSH using the Ellman reagent (0.0198% DTNB in 1% sodium citrate).

### Reactive oxygen species (ROS) formation

To determine the rate of hepatocyte ROS generation during methimazole metabolism, 2′,7′-dichlorofluorescein diacetate (1.6 μM) was added to the hepatocyte incubate. DCFH-DA became hydrolyzed to non-fluorescent dichlorofluorescein (DCFH) in hepatocytes. Dichlorofluorescin then reacted with reactive oxygen species to form the highly fluorescent dichlorofluorescein. 1ml (10^6^ cells) of hepatocytes was taken and the fluorescence intensity of the ROS product was measured using a Jasco FP-750 spectrofluorometer with excitation and emission wavelengths of 500 and 520 nm, respectively [28].

### Data analysis

The results are shown as the Mean±SE for at least three independent experiments. Statistical analysis for the control and experimental groups was performed by a one-way ANOVA (analysis of variance) test. A P < 0.05 was considered as a significant difference.
